# Effect of Indentation Depth on Friction Coefficient in Adhesive Contacts: Experiment and Simulation

**DOI:** 10.3390/biomimetics9010052

**Published:** 2024-01-17

**Authors:** Iakov A. Lyashenko, Thao H. Pham, Valentin L. Popov

**Affiliations:** Department of System Dynamics and Friction Physics, Institute of Mechanics, Technische Universität Berlin, 10623 Berlin, Germany; pham.19@campus.tu-berlin.de (T.H.P.); v.popov@tu-berlin.de (V.L.P.)

**Keywords:** quasi-static tangential and normal contact, indentation, adhesion, elastomer, friction force, shear stress, contact area

## Abstract

The quasi-static regime of friction between a rigid steel indenter and a soft elastomer with high adhesion is studied experimentally. An analysis of the formally calculated dependencies of a friction coefficient on an external load (normal force) shows that the friction coefficient monotonically decreases with an increase in the load, following a power law relationship. Over the entire range of contact loads, a friction mode is realized in which constant shear stresses are maintained in the tangential contact, which corresponds to the “adhesive” friction mode. In this mode, Amonton’s law is inapplicable, and the friction coefficient loses its original meaning. Some classical works, which show the existence of a transition between “adhesive” and “normal” friction, were analyzed. It is shown that, in fact, there is no such transition. A computer simulation of the indentation process was carried out within the framework of the boundary element method, which confirmed the experimental results.

## 1. Introduction

Adhesion refers to the sticking together of solids or of solids and liquids. In biology, adhesion forces play an important role. Due to intercellular adhesion, cells are able to unite into organs, as well as to stick to a substrate [1,2]. Adhesion is actively used by many biological organisms across a wide range of scales—from bacteria, that are adhesively attached to the surfaces [3], to certain amphibians and reptiles capable of moving along inclined surfaces, or even upside down along vertical surfaces, due to adhesion [4,5]. Different animals use various adhesion mechanisms. For example, some insects secrete a fluid with which they attach to surfaces using capillary forces (adhesion due to liquid bridges) [6]. Those capillary forces are sufficient for insects, due to their low body mass. However, this mechanism was proven to be insufficient to adhere larger organisms, such as phyllomedusae or geckos, to surfaces. Therefore, larger animals use an adhesion mechanism based on Van der Waals forces [4,5]. The main reason that we do not often observe adhesion in solid–solid contact is that all solids in nature have rough surfaces and the area of real contact (where the adhesive forces mainly act) is very small compared to the observed area of nominal contact. Animals circumvent this limitation through the use of complex hierarchical adhesive structures on their feet, consisting of large numbers of keratin hairs (setae). On the gecko’s feet, for example, each seta is 2–10 μm wide and about 100 μm long [7,8]. At the end, each seta has branching, protruding structures called spatula (≈200 nm wide and long). When contacting a rough surface, the setae of such paws deform and fill in the roughnesses, whereas the spatulas provide strong adhesive contact. As a result, the real contact area becomes close to the nominal area, which greatly increases the strength of the adhesive contact. In this case, the surface structure of the paw is organized in such a way that the highly adhesive ends do not touch each other, otherwise they would stick together and lose their adhesive properties. Interestingly, a similar structure, consisting of setae, is also used by abalone [9,10], which live in water. It is worth noting that the contribution of Van der Waals forces to adhesive forces in the abalones is only about 20%, while the contribution of vacuum adhesion forces exceeds 60% [10].

For locomotion of biological organisms, attachment and detachment of the adhesive contacts are fundamental [11]. But a much more challenging problem, from a researcher’s point of view, is the influence of adhesion on friction in sliding contacts [12,13,14]. For example, in [14], experiments have been carried out where a spider leg segment has been pulled and pushed along a surface. For some spider species, it has been observed that their attachment system requires sliding in order to form an intimate contact, otherwise the contact area is almost zero. During pushing, the proportion of spatulae in contact were higher than in pulling. Based on the friction experiments, it has been observed that, in the pushing mode, the measured friction force was considerably higher than in the pulling mode. Tangential contact (friction) in adhesive systems is a complicated problem, as adhesion contributions in normal and tangential forces differ considerably in their nature [15].

Adhesion can be controlled by modifying the contact area or the contact strength. Both are dependent on the shear force [15], since pushing the adhesive pad away from the body creates an unstable contact and therefore a decreasing contact area, whereas pulling the pad towards the body leads to an increase in contact area. ”Shear sensitivity” is widespread across climbing animals, including flies, beetles, spiders, leafhoppers, bushcrickets, stick insects, cockroaches, ants, bees, bats, tree frogs and geckos. By shearing the adhesive pads along the surface, these animals can control their attachment to the surface.

In addition to biological organisms, contacts showing enhanced adhesion properties are widely used in a variety of technical applications [16,17,18,19,20]. To understand the processes in adhesive contacts, a large number of both theoretical and experimental investigations are being carried out [15,21,22]. In addition, computer modeling based on modern numerical methods, such as the boundary element methods (BEM), finite element methods (FEM), molecular dynamics (MD), movable cellular automata (MCA), etc., is actively used. However, despite the efforts of many scientific groups, many of the effects observed in adhesive contacts remain incompletely understood. This especially applies to tangential adhesive contacts, which are much more poorly described compared to normal contacts. Understanding the processes occurring in tangential contact is important for understanding what happens in adhesive contact during the movement of biological organisms on inclined surfaces, since such contact is always simultaneously loaded in both normal and tangential directions. In our previous work [23], the propagation of adhesive contacts at indentation and its disappearance at detachment were studied experimentally. The present work is an extension of [23] and investigates the sliding mode (friction) in adhesive contacts under a slowly increasing external load. The materials and experimental techniques are similar to those used in [23].

## 2. Theoretical Estimation of the Friction Coefficient for Adhesive Contacts

In a case where the main contribution to friction is caused by the adhesive interaction between the surfaces, the friction force is approximately given by the equation [24,25,26].

(1) $$F_{x}=\tau_{0}A_{r},$$

where *A_r_* is the real contact area and *τ*_0_ are the tangential stresses that are necessary to break the adhesive bonds and initiate the sliding mode. In typical engineering contacts between solid bodies, usually the real contact area (*A_r_*) is mostly very small compared to the nominal contact area (*A*), due to surface roughness. For instance, in [27], a series of experiments were carried out on the friction between quartz glass and rough copper samples. The result of this work demonstrates that the real contact area (*A_r_*) increases linearly with rising external load on the friction surface (*F_N_*) but still remains small; even at a maximum load of 120 N it was less than 4% of the nominal contact area. Since, in Equation (1), the critical stresses (*τ*_0_) are constant and defined by the adhesive properties of the contacting surfaces and the area, *A_r_* increases linearly with the external force [27]

(2) $$A_{r}=\alpha F_{N}$$

as a result, the well-known Amonton’s friction law emerges:

(3) $$F_{x}=\mu F_{N},$$

where a constant friction coefficient (*μ* = *ατ*_0_) is introduced. Very often, the friction force is more or less accurately described by Amonton’s law (3), especially in the case of dry friction between solid bodies that are not capable of forming strong adhesive contacts. Nevertheless, deviations from Amonton’s law are common, including non-adhesive dry contacts [28].

The other limiting case is friction in contacts that show good adhesion. A special case of such contacts is a pair comprising a hard indenter and a soft elastomer that exhibit macroscopic adhesive properties. The fact is that soft material can be easily deformed so it is able to almost completely fill in the gaps between roughnesses on the surface of a rigid indenter. In the presence of adhesion, such filling of roughness occurs even more successfully due to adhesive forces between the surfaces, especially when the incompressible elastomer acts as the adhesive material. Since the total volume of the elastomer does not change during the deformation, the material extruded locally by the roughness must occupy the new free spaces in the contact, which are the gaps between the rough protrusions on the indenter surface. Therefore, in such adhesive contacts, expression (2) is not valid, since the real contact area (*A_r_*) is very close to the nominal contact area (*A*) (almost full contact is realized). As a result, Amonton’s law (3) becomes inapplicable and the friction law takes the form of [24,25].

(4) $$F_{x}=\tau_{0}A,$$

where *A* is the nominal contact area, which coincides with the real contact area, *A_r_*.

In our previous works [29,30], we studied the tangential contact between steel indenters with radii of *R*~100 mm and the TARNAC CRG N3005 elastomer, which has the elastic parameters *E ≈* 0.324 MPa and *ν ≈* 0.48, determined in [31] by summarizing a large number of experimental data. This type of rubber has distinct adhesive properties, and the friction force in the contact between it and the steel indenter is determined by Equation (4). Note that, depending on the state of the surface in various experiments, the value of stationary stresses (*τ*_0_) in the sliding mode in our experiments ranged from 30 to 60 kPa. In [25], where the authors studied friction between atomically smooth surfaces separated by various organic substances several monolayers thick, it was experimentally shown that in the presence of adhesion, constant tangential stresses are realized in the contact, regardless of the load on the friction surface. Therefore, the regime of “adhesive” friction, given in Equation (4), is observed in both ordinary engineering contacts and in microscopic systems.

According to the classical works [32,33], in adhesive contacts, with an increasing load on the rubbing surfaces (*F_N_*), a transition occurs from the “adhesive” friction mode (4) (in which the friction force (*F_x_*) is proportional to the contact area) to “normal” friction. In the “normal” friction mode, the friction force becomes proportional to the applied load (see Equation (3)). This transition is combined with a rapid decrease in the value of the friction coefficient (*μ*), which is calculated in a standard way

(5) $$\mu =F_{x}/F_{N}$$

with an increase in the normal force (*F_N_*) and its tendency to a constant value (*μ *= const) with a further increase in the load on the contact. It is worth noting that, in adhesive contacts (for which Equation (4) is valid) the value of the formally calculated friction coefficient (*μ*) (5) at low loads (*F_N_*) can be extremely large. This happens because, in adhesive contacts, the resulting normal force (*F_N_*) consists of two oppositely directed components: the elastic reaction force of the support and the adhesive force [34]. If these forces are equal by absolute values, the resulting normal force will be zero at the non-zero contact area. Consider a situation where, simultaneously with normal indentation, tangential movement of the indenter occurs, resulting in a non-zero tangential force (friction force). In this case, in some interval of indentation depth a normal force (*F_N_*) will be close to zero at some finite friction force (*F_x_*), formally leading to infinite values of the friction coefficient, *μ* (5). Note that even negative values of the “friction coefficient” (*μ*) can be realized, if the resulting force is *F_N_* < 0 (for example, in the case of pure adhesive force at positive distances between indenter and elastomer).

The situations with extremely large friction coefficients have been repeatedly observed in our experiments, for instance, in [30], which provides an estimation of the friction coefficient for adhesive contacts formally determined by Equation (5). If we assume that Equation (4) is valid in an adhesive contact and determine the friction coefficient using Equation (5), then we will have

(6) $$\;\mu =\tau_{0}A/F_{N}.$$

If the relationship between the contact area (*A*) and the force (*F_N_*), applied in the normal direction to the friction surface, is known, it is possible to find analytical dependence of the friction coefficient as a function of indentation depth (*d*) or normal force (*F_N_*). It was experimentally shown that, in the presence of tangential motion and a sufficiently large indentation, depth (*d*), even in adhesive contacts, normal force (*F_N_*) and contact radius (*a*) are sufficiently described by the expressions for non-adhesive contact (Hertz contact [35]).

Let us consider the general case of an axially symmetric indenter, where the profile is specified by a power function

(7) $$\;f\left(r\right)=c_{n}r^{n},$$

where *r* is the radial coordinate. Note that the contact problem only has a geometric meaning for *n >* 0, because the function *f*(*r*) must be increasing. When indenting the profile of *f*(*r*) into an elastic half-space, the normal force (*F_N_*) and the contact radius (*a*) are determined by the expressions [36]:

(8) $$\;F_{N}=\frac{2n}{n+1}\times \frac{E^{*}d^{\frac{n+1}{n}}}{{\left(c_{n}\kappa_{n}\right)}^{\frac{1}{n}}},\;a={\left(\frac{d}{c_{n}\kappa_{n}}\right)}^{\frac{1}{n}}$$

where the following functions are introduced

(9) $$\kappa_{n}=\frac{\sqrt{\pi }}{2}\times \frac{n\Gamma \left(\frac{n}{2}\right)}{\Gamma \left(\frac{n+1}{2}\right)},\;E^{*}=\frac{E}{1-\nu^{2}}$$

where Γ(∙) is a standard gamma function [37]

(10) $$\Gamma \left(n\right)∶=\int_{0}^{\infty }t^{n-1}e^{-t}dt$$

For axially symmetric contacts, the area of the contact can be found as *A = πa*^2^. For such a case, from Equation (6) we will have

(11) $$\;\mu ={\pi a^{2}\tau }_{0}/F_{N}.$$

In the case where the friction force is proportional to the contact area (*F_x_ = τ*_0_*A*) (4), the friction coefficient (*μ*) is determined by Equation (11), which, when using expressions (8)–(10), leads to the dependence of the friction coefficient (*μ*) on the indentation depth (*d*)

(12) $$\mu (d)=\frac{\pi \tau_{0}(n+1)}{2nE^{*}{\left(c_{n}\kappa_{n}\right)}^{\frac{1}{n}}}\times \frac{1}{d^{\frac{n-1}{n}}},$$

or the same as a function of normal force (*F_N_*)

(13) $$\;\mu (F_{N})=\pi \tau_{0}{\left(\frac{n+1}{2nE^{*}c_{n}\kappa_{n}}\right)}^{\frac{2}{n+1}}\times \frac{1}{{\left(F_{N}\right)}^{\frac{n-1}{n+1}}}.$$

Note that the estimates (12) and (13) are valid only for sufficiently large indentation depths, at which the influence of adhesion on the contact size becomes negligible.

There are some important conclusions following from Equation (13):

(1) In the case of 0 *< n <* 1, the friction coefficient (*μ*) increases with the increase in normal force. The reason for this behavior lies in the fact that with such forms of the indenter, the normal force (*F_N_*) increases slower than the contact area (*A*).
(2) For conical indenters, where *n *= 1, the friction coefficient does not depend on either the normal force (*F_N_*) or the indentation depth (*d*) and is equal to a constant value

(14) $$\;\mu_{conical}=\frac{2\tau_{0}}{E^{*}c_{n}}.$$

This is a very important point, since it means that in experiments with conical indenters we will have a constant friction coefficient, despite the fact that the friction force, as before, is given by the equation *F_x_ = τ*_0_*A* (4). Therefore, experiments with conical indenters can potentially be misinterpreted, and when analyzing their results, it is necessary to pay additional attention to this feature.

(3) In the case *n* > 1, the friction coefficient decreases with the increase in normal force (*F_N_*).
(4) In the case when *n >>* 1, the indenter turns into a flat stamp (the contact area (*A*) is a constant and is independent from indentation depth and normal force). Therefore, for *n* → ∞, Equation (13) shows the asymptotic behavior

(15) $$\mu \propto \frac{1}{F_{N}}.$$

When choosing the parameter $c_{n}=a_{0}^{1-n}$ in Equation (7), while *n* → ∞, the profile *f*(*r*) describes a cylinder with a flat base with a radius of *a*_0_. 

In the present work, we have investigated adhesive contact between a spherical indenter and an elastomer. For a small indentation depth, a sphere can be approximated well by a parabolic shape. The expression (12) for the case of a parabolic indenter with *n *= 2 and *c_n_* = 1/(2*R*) is reduced to the equation previously used in [30] in the form

(16) $$\;\mu \left(d\right)=\frac{3\pi \tau_{0}}{4E^{*}}\sqrt{\frac{R}{d}}.$$

According to (16), the friction coefficient (*μ*) decreases with an increase in the indentation depth (*d*). The second Equation (13) for the particular case of the parabolic indenter will take the form

(17) $$\;\mu \left(F_{N}\right)=\pi \tau_{0}{\left(\frac{3R}{4E^{*}}\right)}^{\frac{2}{3}}\frac{1}{{\left(F_{N}\right)}^{1/3}}.$$

Equation (17) was previously used in [38,39] to describe friction between surfaces coated with thin layers of soft plastic materials. If there is a thin layer of material in the contact zone that is subject to plastic flow, a situation similar to adhesive friction is observed, since the friction force is also given by the equation *F_x_ = τ*_0_*A* (4), where the stress *τ*_0_ has the meaning of tangential critical stress of plastic flow of the thin layer of material.

Let us note an important point. Formally, Equations (16) and (17) show a constant decrease in the friction coefficient (*μ*) as the indentation depth (*d*) or normal force (*F_N_*) increases. Therefore, if the expressions (16) and (17) turn out to be valid at extremely high loads, then the friction coefficient at high loads should be quite small. However, such an effect has nothing to do with a change in the friction mechanism, as is observed, for example, when establishing a superlubricity mode with a negligible friction coefficient [40]. In (16) and (17), the nature of the friction coefficient reduction is related to the contact geometry during the indentation of a spherical (parabolic) indenter. During indentation, the contact area (*A*) increases slower than the corresponding normal force (*F_N_*), which, according to the general Equation (6), leads to a decreasing friction coefficient.

**Figure 1:**
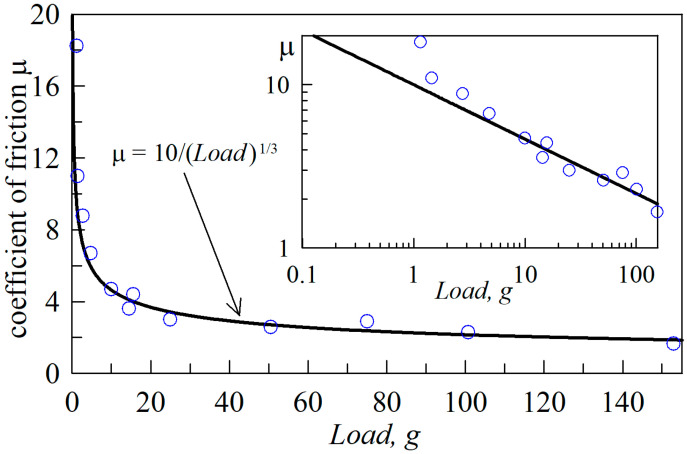
Dependence of the friction coefficient (*μ*) on load. Symbols: experimental data from [32]. Solid line: approximation (17). The inset to the figure shows the same dependence in logarithmic coordinates.

Now let us return to the results of [32,33] that describe the transition between the modes of “adhesive” (4) and “normal” (3) friction with an increase in the external load *F_N_* on the friction surface. In these works, the presence of such a transition is explained as a decrease in the friction coefficient (*μ*) as normal force (*F_N_*) increases, and its tendency towards a constant value if *F_N_* becomes large enough. In [32], the results of an experiment were described in which the friction of steel on indium was studied in a load range from 2 to 150 g. The work states that for loads greater than 100 g the friction coefficient does not change and is *μ* ≈ 2, while for small loads of the order of 2 g, the friction coefficient exceeds 18. In Figure 1, the symbols show experimental data from [32], on the basis of which the authors draw such conclusions. Note that, here, the maximum load of 150 g corresponds to a normal force of *F_N_* ≈ 1.5 N.

In addition to the experimental data, Figure 1 shows dependence (17) which, as it turns out, describes the dependence obtained in the experiment well. The important point here is that Equation (17) is an expression of “adhesive” friction, in which the friction force is proportional to the contact area (*F_x_ = τ*_0_*A*) (4). This means that (17) does not imply a transition between the “adhesive” and “normal” friction modes. And, the illusion of a transition lies in the fact that the power function (17) decreases quite slowly, as can be seen in the inset of the figure, where the same dependencies are presented in logarithmic coordinates. Thus, we can conclude that the experimental data from [32] do not confirm the presence of a transition between friction modes; furthermore, to test the hypothesis about the existence of such a transition, it is necessary to conduct additional experiments, which are described in the following sections of this paper.

## 3. Experimental Technique

### 3.1. Description of Experimental Equipment

**Figure 2:**
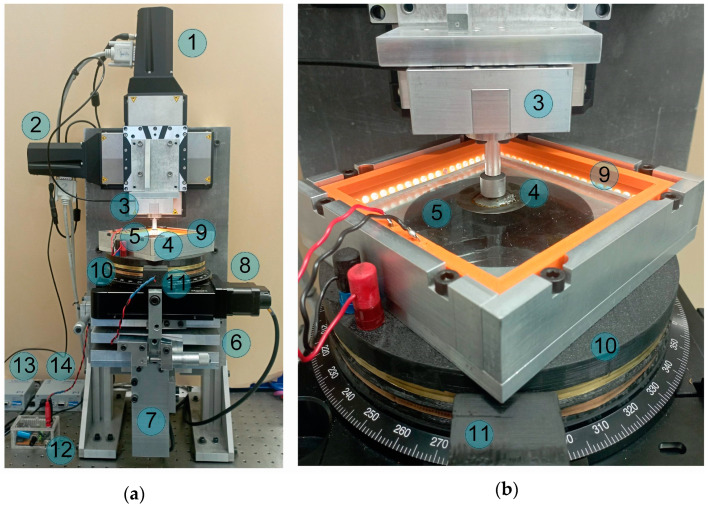
(**a**) a general view of the experimental setup; (**b**) close-up view of the contact area between the spherical indenter (4) and the elastomer (5) illuminated by a surrounding LED light (9), powered by a system of sliding contacts (10) and (11).

All experiments described in this paper have been carried out with the experimental setup shown in Figure 2. The left panel shows a general view of the device, while the right panel shows the contact area. In both panels, the main elements of the system are indicated by numbers. A spherical steel indenter (4) is attached to a three-axis ME K3D60a force sensor (3), which operates over the force range of ±100 N. Note that in our previous works (see, for example, [31,41]) we used the ME K3D40 sensor, which operates in a smaller range of ±10 N. The force sensor (3) measures all three components of normal force. The electrical signal from the sensor (3) is amplified using a 4-channel amplifier GSV-1A4 SUBD37/2, equipped with three channels. The amplified signal is transferred to a computer using an NI USB-6211 16–bit ADC. The indenter (4) is moved in the normal and tangential directions using connected high-precision linear stages PI L-511.24AD00 (1) and (2). These drives are controlled by USB controllers PI C-863, which are shown in Figure 2, in positions (13) and (14).

Sheets of transparent rubber TARNAC CRG N3005 and CRG N0505 with thicknesses *h *= 5 mm are used as elastomers (5), which are indented using a steel counter body. The almost perfect transparency of these elastomers allows direct observation of the contact area, which is carried out with the Ximea 2.2 MP MQ022CG-CM digital camera using a FUJINON HF16SA-1, 2/3” lens. The camera is in position (7) and is blocked from the observer by a fixed aluminum plate. The elastomer CRG N3005 has an elastic modulus *E ≈* 0.324 MPa [31], while the elastic modulus of the indenter material (steel) is *E ≈* 2 × 10^5^ MPa, which is 5 orders of magnitude higher. Therefore, when analyzing the experimental data, the indenter is considered as absolutely rigid. The second material used in this paper, CRG N0505, is even softer.

The tilting mechanism (6) allows a manual change of the position of the elastomer in the horizontal plane, which is important when conducting experiments with tangential motion described below. The 8MR190-90-4247-Men1 motorized rotation stage (8), controlled by the 8SMC5-USB-B8-1 USB controller, adds an elastomer rotation option to the setup, which is also used in the experiments described below.

In a previous paper [41], the setup shown in Figure 2 was described in detail, including various modifications. However, an additional modification was added to the device used in this work, which was not present in [41]. This refers to the system of sliding contacts (10) and (11). This system enables an uninterrupted power supply from the regulated power supply unit (12) to the LED lighting frame (9), providing stable lightning in experiments with circular motion when the rotation stage is activated. Homogeneous qualitative illumination of the contact area is of great importance as we process photographs of the contact area in order to find the magnitude of the contact area and also to observe the dynamic processes occurring in the contact due to the rearrangement of the contact boundary; the lightning (9) consists of 80 white LEDs (20 from each side).

**Figure 3:**
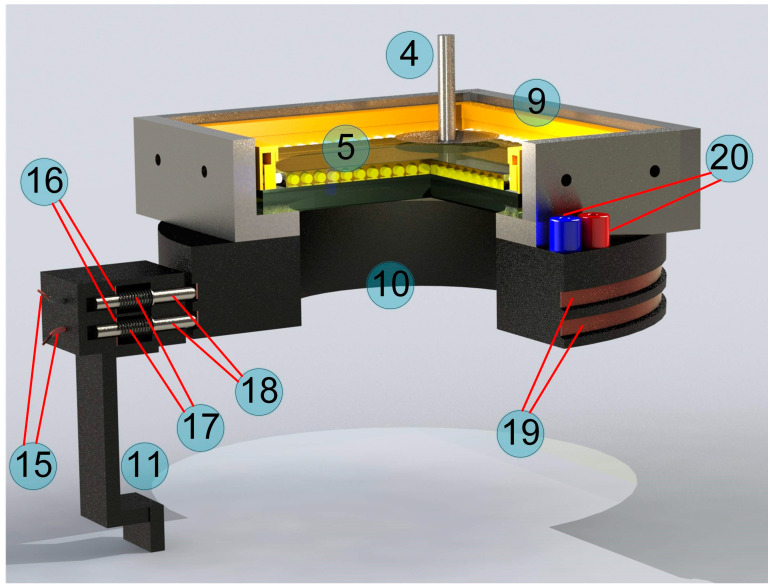
An accurate 3D model showing the sliding contact mechanism indicated as numbers (10) and (11) in Figure 2.

Since the developed sliding contact system is a new improvement to the existing equipment, which has not been described by us before, we will elaborate on it in more detail. Figure 3 provides a more detailed view of the system under discussion, separately from the installation.

Here, the contact system is shown in section view, which allows us to trace the path of electrical energy transfer from the power supply unit (12) to the light frame (9). For convenience, Figure 3 shows the numbered elements, which are the same as used above in Figure 2; the new elements in Figure 3 continue the numbering order of Figure 2. Note that Figure 3 shows not a real photograph, but an accurate 3D model preserving all proportions. This 3D model was used to manufacture the installation elements (10) and (11) that were printed out with the “QIDI TECH i Fast FDM 3D Printer”. The electric current from the power supply (12) is transferred to the brass foil plates (16) via cables (15). These plates are in contact with the steel pins (18) through springs (17). Through contact, the pins transmit an electrical current to the metal rings (19), which are electrically connected to the sockets (20). The light frame (9) is connected to the sockets (20) (see Figure 2b). The springs (17) are needed to press the pins (18) against the conductive strips (19) to ensure stable electric sliding contacts. A total of 4 pins are used in the system to ensure reliable contact: 2 for each contact strip. In this case, the cross-section in Figure 3 shows only two pins, each corresponding to different contact strips. Note that the metal strips (19) in the real photograph in Figure 2 have different colors as they are made of different materials: brass (top) and copper (bottom). Initially, different materials were used to determine which was best suited to provide a reliable electrical contact. However, since both materials showed good contact properties, there was no need for further modification of the sliding contact pairs.

### 3.2. Techniques of Experimentation

**Figure 4:**
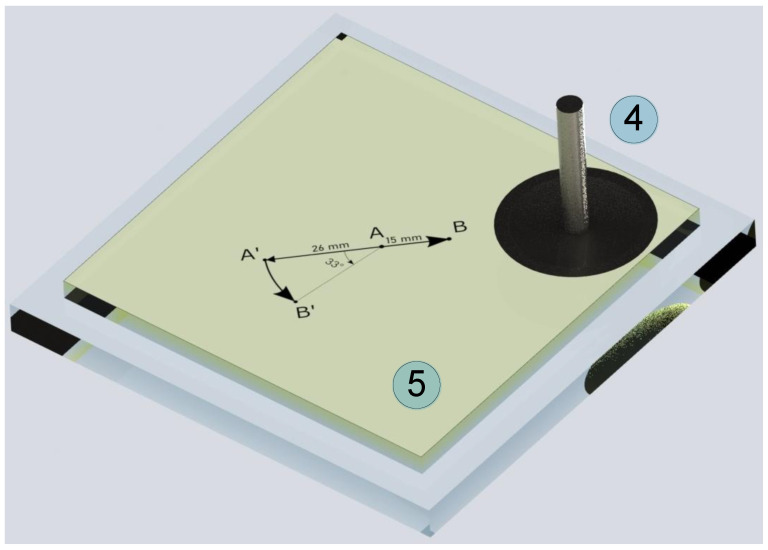
Schematic drawing of the experiment where the indenter (4) is in contact with the elastomer (5). The path of the indenter is shown by trajectories *AB* and *A’B’*. A spherical indenter with a radius of *R *= 100 mm is shown to allow visual assessment of the relationships between the quantities displayed in the figure and the indenter radius.

Since the purpose of the present work was to determine the characteristics of tangential contact in a quasi-static mode, the indenter had to be moved at low velocities so that the relaxation process could take place in the elastomer during the movement. In [42], it was shown that the contact between a rigid indenter and the materials we used as elastomers can be considered quasi-static, when the indenter velocity does not exceed *v* = 5 μm/s. The main goal of the present work was to study the dependence of the friction coefficient in adhesive contact on the load on frictional surfaces. For this purpose, a series of experiments were carried out under two different indentation scenarios. In both scenarios, the indenter was first moved to the elastomer in a purely normal direction until first contact was established. After the indenter touched the elastomer surface, in the first indentation scenario, simultaneous movement in the normal and the tangential directions began while the elastomer remained stationary. In this case, the velocity of the indenter in the tangential direction was *v_t_* = 5 μm/s, while the velocity in the normal direction was much lower at *v_n_* = 0.2 μm/s. Therefore, the indentation angle was α = arctan (0.2/5) ≈ 2.29°. In our previous work [23], we have experimentally shown and theoretically justified that when the indentation angle is α *<* 20°, the contact properties are very close to the tangential contact. Here we indent at a much smaller angle of 2.29°, so we can consider that at each moment the conditions of tangential contact are realized. However, the indentation depth (*d*) and the normal force (*F_N_*) increase with time, which allows us to analyze the dependence of the friction coefficient (*μ*) on these values. The described scenario is illustrated by trajectory *AB* in Figure 4, which shows an elastomer (5) layer on a glass plate.

The second scenario was gradual indentation with the same normal velocity (*v_n_* = 0.2 μm/s) without tangential shift of the indenter, but with rotation of the elastomer using a rotation stage. In this case, the angular velocity of the rotation stage was chosen based on the assumption that the relative tangential shear velocity of the indenter at the center of the contact relative to the elastomer surface should be the same (5 μm/s) as in the first scenario. In these experiments, the indenter was located at a distance of *L *= 26 mm from the center of rotation of the elastomer. To ensure that the indenter velocity relative to the elastomer is *v_t_* = 5 μm/s, the angular velocity is determined as follows

(18) $$\dot{\varphi }=\frac{v_{t}\times 180}{\pi L}=\frac{5\times 10^{-6}\times 180}{26\times 10^{-3}\pi }\approx 0.011\;\left({}^{\circ}/s\right).$$

The indenter trajectory in this scenario is shown in Figure 4 as *A’B’*. To ensure that in the experiment with elastomer rotation the indenter passes the same path as in the experiment with linear motion along the trajectory *AB*, the equality *AB = A’B’* must be satisfied, which is obtained when the elastomer is rotated by the angle of

(19) $$\varphi =\frac{A^{\prime }B^{\prime }\times 180}{\pi L}=\frac{15\times 180}{26\pi }\approx 33{}^{\circ}.$$

In both experiment scenarios, after reaching the maximal indentation depth (*d*_max_), the indenter was pulled off in a normal direction at the velocity *v_n_* = 1 μm/s without tangential motion, until the contact was completely broken.

## 4. Results of Experiments

### 4.1. Two Scenarios of Contact Loading

**Figure 5:**
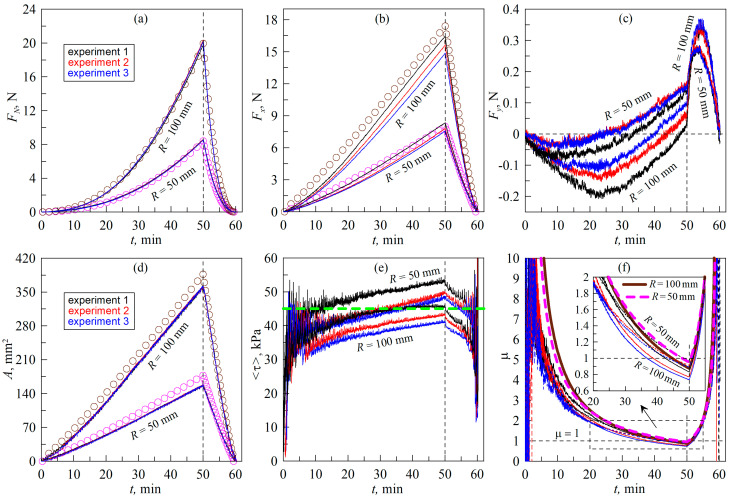
Dependences of the normal (*F_N_*) (**a**), tangential (*F_x_*) (**b**) and lateral (*F_y_*) (**c**) contact forces, contact area (*A*) (**d**), average tangential stress $\langle \tau \rangle ={\left(F_{x}^{2}+F_{y}^{2}\right)}^{1/2}/A$ (**e**) and formally defined friction coefficient $\mu ={\left(F_{x}^{2}+F_{y}^{2}\right)}^{1/2}/F_{N}$ (**f**) on time (*t*). In the figure, the dependencies corresponding to the indenters with radii of *R *= 100 mm and *R *= 50 mm are labeled. The figure corresponds to the experiment with linear motion of the indenter at resting elastomer, the experiment layout is shown using trajectory *AB* in Figure 4. A Supplementary Video is also available for the figure (Video S1).

In Figure 5, the lines show the results of the experiments carried out according to the scenario shown by trajectory *AB* in Figure 4, i.e., here, the indenter was slowly immersed into the elastomer while moving simultaneously in the normal and tangential directions. The figure shows the results of six experiments, three experiments in a sequence for indenters with radii of *R *= 100 mm and *R *= 50 mm when they were indented into a layer of TARNAC CRG N3005 rubber with a thickness *h *= 5 mm. Three experiments (shown in different colors) were performed for each indenter to demonstrate the reproducibility of the experiments.

In all experiments, the indenters were pressed to a maximum depth of *d*_max_ = 0.6 mm, which at a normal speed of *v_n_* = 0.2 μm/s requires an indentation time of *t* = 50 min. During this time, the indenter had time to pass a distance of *L *= 15 mm in the tangential direction, since its tangential velocity was *v_t_* = 5 μm/s. After reaching the maximum indentation depth (*d*_max_) (this time moment is shown in all panels of Figure 5 by vertical dashed lines), the indenter was pulled off in the normal direction at a velocity of *v_n_* = 1 μm/s until the contact was completely lost.

**Figure 6:**
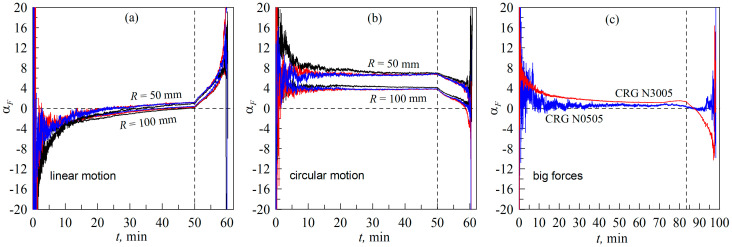
Dependences of the angles *α_F_* (21) between the directions of the resulting tangential force (*F_t_*) and the indenter trajectory: (**a**) in the experiment with linear motion, the results of which are shown in Figure 5; (**b**) in the experiment with elastomer rotation; (**c**) in the experiment with different elastomers.

In light of the dependencies shown in Figure 5, it follows that, for both indenters (*R *= 100 mm and *R *= 50 mm), both the normal force (*F_N_*) and the friction force (*F_x_*) increase over time. The increase in friction force is due to the tangential loading of the contact as well as the increase in the contact area (*A*) due to the monotonic increase in the indentation depth (*d*). Note that in the experiments there is always a force component perpendicular to the direction of the movement of the indenter. This force is denoted as *F_y_* and is shown in Figure 5c. The lateral force (*F_y_*) arises due to the presence of inhomogeneities on the elastomer surface, deviations of the geometric shape of the indenter from the spherical one, etc. The presence of lateral force leads to the fact that the tangential force acting in the contact is not *F_x_*, directed opposite to the direction of motion, but

(20) $$F_{t}=\sqrt{F_{x}^{2}+F_{y}^{2}},$$

directed at an angle

(21) $$\alpha_{F}=arctan\frac{F_{y}}{F_{x}}$$

to the direction of the movement of the indenter. In experiments in which there is no lateral movement of the indenter, the lateral force (*F_y_*) should be much smaller than *F_x_*, while the angle *α_F_*, determined by Equation (21), should be close to zero. In the present paper, the behavior of the friction coefficient (*μ*) is studied, and it is known that it can depend on the evolution of the ratio between the magnitudes of the tangential and lateral forces (21), as was shown in [43]. Therefore, for completeness, Figure 6a shows the dependences of the angle *α_F_*(*t*) for all experiments, the results of which are shown in Figure 5. Based on this figure, the angle *α_F_* takes on large absolute values only at the initial stage of indentation and in the detachment phase, while in the main indentation phase this angle does not exceed the value of a few degrees.

In all experiments, three force components and the time dependencies of the contact area (*A*) shown in Figure 5d were calculated. The contact area is determined directly from the experiment, since transparent rubber is used as elastomer and the contact area is clearly visible (see the description in Section 3 of the paper, as well as all Supplementary Videos). The availability of information about the contact area allows us to calculate the dependencies of mean tangential stresses *<τ>*. Since the lateral force (*F_y_*) is present in the experiments, for greater accuracy in presenting the results, the tangential stresses *<τ>* and the coefficient of friction (*μ*) were determined as

(22) $$\langle \tau \rangle =\frac{F_{t}}{A}=\frac{\sqrt{F_{x}^{2}+F_{y}^{2}}}{A},\;\mu =\frac{F_{t}}{F_{N}}=\frac{\sqrt{F_{x}^{2}+F_{y}^{2}}}{F_{N}}.$$

exactly these dependencies are shown in Figure 5e,f.

Note that Figure 5e shows a small but monotonic increase in shear stresses *<τ>* throughout the tangential motion (before the vertical dashed line, after which detachment of the indenter in the normal direction was performed). Previously, in numerous experiments we conducted on tangential contact with a fixed indentation depth (*d*_max_), the stresses *<τ>* always decreased slightly with time. We interpreted such a decrease as a decrease in the adhesive forces between the surfaces due to their oxidation, contamination, etc., during the friction process [29,30]. However, in a previous work [23], which studied the effect of indentation angle on contact processes, a slight increase in stress *<τ>* with the increase in indentation depth was also observed at small indentation angles (when the contact is close to tangential). Such an increase can be due to the influence of additional friction on the contact boundary line. In our recent work [29], it was suggested that friction force in adhesive contacts has two main contributions. The first contribution is the friction from the contact area: *F*_area_ = *τ*_0_*A* (4). The second contribution is the friction at the boundary line, *F*_boundary_ = *qD*, where *D* is the width of the contact facing the direction of motion and *q* is the linear force density. Total friction force is the sum of these two components:

(23) $$F_{total}=qD+A\tau_{0},$$

or, for mean shear stresses, *<τ>* = *F*_total_/*A*:

(24) $$\langle \tau \rangle =\frac{qD}{A}+\tau_{0}.$$

Actually, in the experiment, we measure <*τ*> and not *τ*_0_, which should be a constant. The results shown in Figure 5e show an increase in shear stresses with an increase in contact area. After the vertical dashed line, the contact area decreases during the pull-off phase in the normal direction, and the value of <*τ*> decreases too. This means that we unambiguously observe here an increase in shear stresses with an increase in contact area, the same was observed in our previous work [23] and in further experiments in this work. One possible explanation of such a behavior can be determined using Equation (24), if with an increase in the contact area the ratio *D*/*A* also increases. As shown in [29], for the circular contact area, ratio *D*/*A* decreases with the increase in contact area. But in our experiments, complicated propagation of the contact line was observed; the contact boundary line was rough and it significantly changed the values of *D* in comparison with the perfectly circular contact. We observed an increase in shear stresses with the increase in contact area, which should be investigated in more detail; however, that is outside of the scope of the present work because it needs an additional complex analyzation of the form and length of the contact boundary line, which can also have a fractal character. However, based on the data shown in Figure 5, we can state that the discussed increase in *<τ>* is insignificant and with sufficient accuracy the relation *F_x_ ≈ τ*_0_*A* (4) can be used. Moreover, in the case we are now considering, in all experiments, *τ*_0_ *≈* 45 kPa (this value is shown in Figure 5e by the horizontal dashed line).

Note that the stresses denoted as *<τ>* in Figure 5e remain nearly constant during indenter sliding; despite this fact, the normal force (*F_N_*) varies over a large range. Thus, Figure 5a shows that, for an indenter with a radius of *R *= 100 mm, the maximum force was of the order of 20 N, which is much greater than the maximum load of 1.5 N in the classical experiment performed by McFarlane and Tabor [32], the results of which are shown in Figure 1. However, despite the significantly higher normal load values, the friction coefficient (*μ*)*,* shown in Figure 5f, decreases throughout the whole indentation. This experimentally confirms that, in the whole range of normal forces, the relation *F_x_ ≈ τ*_0_*A* (4) is fulfilled and the transition to “normal” friction, *F_x_ = μF_N_* (3), does not take place. Note that at the beginning of indentation, at low normal forces, large fluctuations of both normal and tangential forces are observed. This is caused by the irregularities that are inevitably present on the surfaces of the rubber and the indenter. At small indentation depths (*d*), inhomogeneities strongly influence the contact configuration and contact forces, especially in the presence of adhesion. Moreover, at the very beginning of indentation (when the indenter just touches the elastomer surface), the normal force can often pass through zero value, changing its sign. As a result, the friction coefficient (*μ*) changes dramatically over a wide range of values at the beginning of indentation. However, once the normal force acquires a steadily increasing trend, the friction coefficient (*μ*) begins to decrease monotonically, as confirmed by Equation (17).

In addition to the experimental results, as depicted in Figure 5 and displayed by solid lines, the results of the boundary element method (BEM) simulation are shown with symbols. This method allows us to calculate the dependencies of the normal force (*F_N_*) and the contact area (*A*) on the indentation depth (*d*) when immersing an indenter with an arbitrary geometrical profile (in our case it is a sphere with radius *R*) into an elastic layer with a fixed thickness (*h*) in the normal direction. The need for modeling lies precisely in the limited thickness of the elastomer *h*, since in this case the analytical estimates (16) and (17) for the half-space become incorrect. The BEM algorithm is described in [44], and its experimental verification was performed in [31]. In the simulation, the results of which are shown in Figure 5, the elastomer parameters *E *= 0.324 MPa, *ν *= 0.48, *h *= 5 mm, corresponding to the TARNAC CRG N3005 material sheet [31], were used, with the indenter radii being exactly the same as the experimental ones. In the experimental work [30], it was shown that, at the indentation stage in the considered case, a low value of the specific work of adhesion (Δ*γ* = 0.0175 J/m^2^) was realized, which was then used in the simulation of normal indentation. With such a low value of Δ*γ*, the influence of adhesion is very weak, which has been observed previously in many experiments and is associated with the influence of the roughness [45] that prevents the contact from propagating. It should be noted that, at the detachment stage, Δ*γ* is already much higher and amounts to Δ*γ* = 0.35 J/m^2^. But such a large value of Δ*γ* can be realized during the pull-off phase in a purely normal direction when the contact is not tangentially loaded (see, for example, [46], where very similar value of Δ*γ* = 0.326 J/m^2^ was found). Note that the value of Δ*γ* is not stable and strongly depends on the duration of the contact, contact pressure (indentation depth), roughness of contact surfaces, their chemical properties and many other parameters [42,47].

In the experiment described in the proposed work, there is always tangential loading in the contact, including the final stage of indenter detachment when it moves in a purely normal direction. Therefore, to simplify the consideration for both indentation and detachment, the same value of Δ*γ* = 0.0175 J/m^2^, which corresponds to a nearly adhesion-free contact, was used in the simulations.

In Figure 5a, the symbols show the normal force dependences of *F_N_*(*t*) obtained in the simulation. One important detail is worth mentioning here. In the experiment, the maximum indentation depth was *d*_max_ = 0.6 mm, but BEM modeling shows that, for this depth, a greater value of normal force is needed than that which was experimentally observed. The problem is that, in a real experiment, there are irregularities and roughnesses on the indenter and elastomer surfaces and the indentation depth in the experiment is counted from the moment of the first contact, which can occur between the protrusions on the surfaces. In this case, the true indentation depth (*d*) between the indenter and the elastomer will be less than what was recorded in the experiment. Such problems are often observed in experiments with spherical indenters that are conducted under conditions of controlled movement (fixed grips). Therefore, in the simulation, the maximum indentation depth (*d*_max_) was chosen so that it approximately corresponds to the maximum normal force (*F_N_*) observed in the experiment. In the case shown in Figure 5, the maximum depth (*d*_max_) in the simulation was 0.568 mm. This value is only 0.032 mm smaller than in the experiment, which is within the experimental accuracy of determining the first point of contact.

The dependencies of *F_N_*(*t*) and *A*(*t*), shown by symbols in Figure 5a,d, are obtained directly from BEM simulations of the normal indentation process. The dependence of *F_x_*(*t*), corresponding to the symbols in Figure 5b, is the result of a simulation-based calculation where the friction force is defined as *F_x_ = τ*_0_*A* (4). In this formula, the contact area (*A*) is the result of BEM, and the used stress value *τ*_0_ = 45 kPa is shown in Figure 5e by the horizontal dashed line, being an average value that is observed experimentally. Since we now have the simulation-compliant dependencies *F_N_*(*t*) and *F_x_*(*t*), it is easy to calculate the theoretical dependencies of the friction coefficients as *μ = F_x_/F_N_* (5). These *μ*(*t*) dependencies are shown in Figure 5f, for the indenter with radius *R *= 100 mm in solid and for the indenter with radius *R *= 50 mm in dashed bold lines. It can be seen from Figure 5f that the simulation results adequately describe the experiment and further confirm that there is no transition between the “adhesive” (4) and “normal” (3) friction modes. Another interesting feature is that the theoretical dependencies of *μ*(*t*) shown in the graph have common points (intersection points), at *t* ≈ 22.9 s during the indentation phase and at *t* ≈ 54.9 s during detachment. At the same time, at small indentation depths (*d* increases linearly with *t*), an indenter with a radius of *R *= 100 mm shows higher values of the friction coefficient (*μ*) compared to an indenter with a radius of *R *= 50 mm, which qualitatively agrees with the analytical estimate for a half-space (16). However, when the depth (*d*) exceeds the critical value, the situation is reversed. At the point of the intersection of dependences at some fixed value of *d*, both indenters show the same value of the friction coefficient (*μ*). In the experiment, a similar situation is observed, since the experimental dependences of *μ*(*t*) for indenters with different radii (*R*) also have common intersection points. This suggests that elastomer layers with limited thickness show a more complex behavior than the in the case of a half-space, for which, according to Equation (16), and all other things being equal, an indenter with a larger radius always shows a higher coefficient of friction.

**Figure 7:**
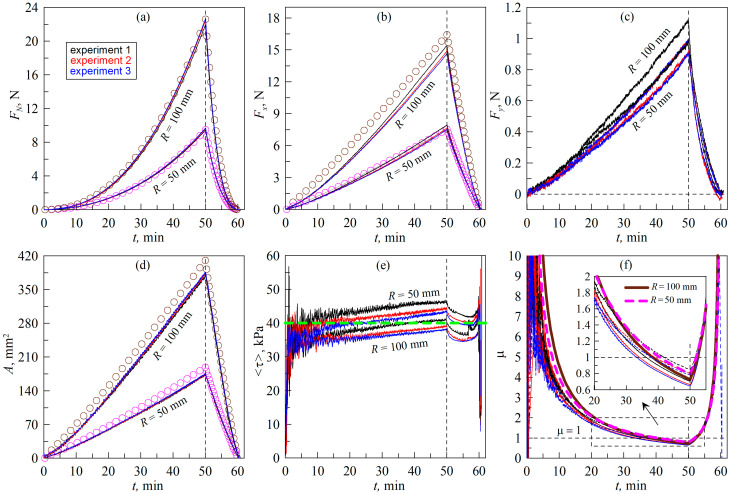
Dependences of the normal (*F_N_*) (**a**), tangential (*F_x_*) (**b**) and lateral (*F_y_*) (**c**) contact forces, contact area (*A*) (**d**), average tangential stress $\langle \tau \rangle ={\left(F_{x}^{2}+F_{y}^{2}\right)}^{1/2}/A$ (**e**) and formally defined friction coefficient $\mu ={\left(F_{x}^{2}+F_{y}^{2}\right)}^{1/2}/F_{N}$ (**f**) on time (*t*). In the figure, the dependencies corresponding to the indenters with radii of *R *= 100 mm and *R *= 50 mm are labeled. The figure corresponds to the experiment with torsion of the elastomer when the indenter moves only in the normal direction. The layout of the experiment is shown using trajectory *A’B’* in Figure 4. A Supplementary Video is also available (Video S2).

Figure 7 shows the results of an experiment in which the indenter was moved only in the normal direction and the elastomer is rotated using the rotation stage (8) shown in Figure 2. The scenario of such an experiment is shown in Figure 4 with trajectory *A’B’*.

In this case, it is possible, in principle, to realize a quasi-infinite motion, since the elastomer can be rotated around its axis for any length of time; the detailed technic of the experiment is described in Section 3.2 of the paper. The basic idea is that, with a sufficiently large distance of the indenter from the rotation axis of the elastomer, we should obtain the equivalent of linear indenter motion. Therefore, in the experiment the indenter was moved to the maximum possible distance from the rotation axis: *L *= 26 mm. Figure 7 shows dependencies similar to those shown above in Figure 5, so we will focus on describing the differences in the results obtained. These are:

(1) In the case of the elastomer rotation (Figure 7), the maximum indentation depth (*d*_max_ = 0.6 mm) corresponds to a larger normal force (*F_N_*) and contact area (*A*) compared to the case of the tangential indenter movement (Figure 5). This can be explained by the fact that, in the experiment shown in Figure 7, the initial contact of the indenter with the elastomer was carried out in the area where there were no pronounced irregularities. This is confirmed by the fact that the BEM simulation results, shown in Figure 7, also correspond to a maximum depth of *d*_max_ = 0.6 mm, while in Figure 5, in the simulation, the maximum depth (*d*_max_) has a smaller value of 0.568 mm (see description of Figure 5 in the text of the paper).
(2) In Figure 7, smaller values of tangential stresses (*τ*_0_) are observed. Therefore, the value *τ*_0_ = 40 kPa was chosen in the BEM simulation, while in the situation demonstrated in Figure 5, a higher value of *τ*_0_ = 45 kPa was used. It is worth noting here that the steady-state value of *τ*_0_ may differ slightly in different experiments [29,30] since it depends on the current chemical state of the friction surfaces, which changes with time. Therefore, the existence of such differences is not related to the geometric features of the compared experiments.
(3) The objectively registerable difference between the experiment with rotation of the elastomer (Figure 7) and the experiment with tangential movement of the indenter (Figure 5) is that, in the case of rotation of the elastomer, larger values of the lateral force (*F_y_*) are observed for obvious reasons (see Figure 7c). This causes the direction of the resulting force (*F_t_*) to change, as shown in Figure 6b. However, this fact does not affect the dependence of the friction coefficient *μ*(*t*), which is shown in Figure 7f (compare with Figure 5f).
(4) In the experiment with elastomer rotation, visible differences are observed in the contact configuration and in its evolution compared to the experiment using linear motion. These differences can be seen in the Supplementary Videos attached to the article. These differences are also due to the fact that, when the elastomer is rotated, the parts of the indenter that are in contact have different distances from the rotation axis and therefore slide with different linear velocities through the elastomer. The differences in slip velocities can be significant as the contact size increases. This feature opens up the possibility of analyzing the influence of movement velocity on the sliding processes in experiments using a fixed angular velocity for the elastomer. Moreover, rotating contacts are often used in various branches of technology, so such studies are also of practical interest.

In general, despite the differences mentioned above, the data shown in Figure 5 and Figure 7 are largely equivalent, demonstrating that both loading scenarios (see Figure 4) can be used in experiments to study tangential contact.

### 4.2. Friction Coefficient at High Loads

**Figure 8:**
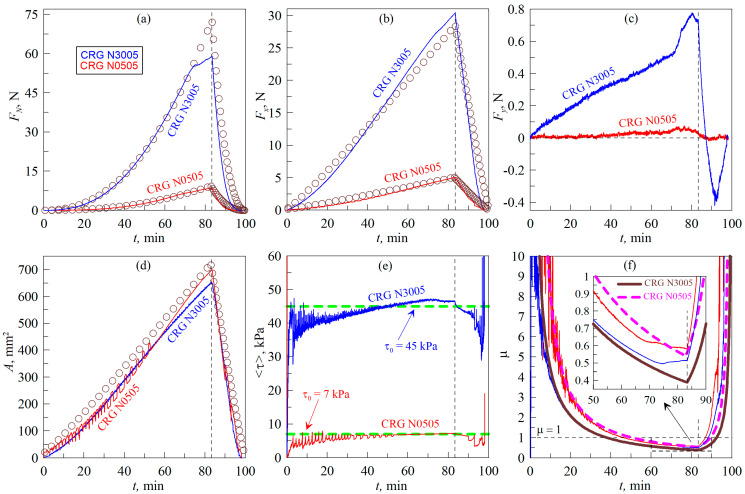
Dependences of the normal (*F_N_*) (**a**), tangential (*F_x_*) (**b**) and lateral (*F_y_*) (**c**) contact forces, contact area (*A*) (**d**), average tangential stresses $\langle \tau \rangle ={\left(F_{x}^{2}+F_{y}^{2}\right)}^{1/2}/A$ (**e**) and formally defined friction coefficient $\mu ={\left(F_{x}^{2}+F_{y}^{2}\right)}^{1/2}/F_{N}$ (**f**) on time (*t*). In the figure, the dependencies corresponding to the elastomers CRG N3005 and CRG N0505 are labeled, and the indenter radius in both experiments was *R *= 100 mm. In panel (d), only the result of BEM simulations for material CRG N0505 is shown, because dependence for CRG N3005 is almost the same. The figure corresponds to the experiment with tangential displacement of the indenter, the layout of which is shown using trajectory *AB* in Figure 4. A Supplementary Video is available for the figure (Video S3).

The experimental results described in Section 4.1 show that a decrease in the formally calculated coefficient of friction is observed over the entire *F_N_* load range. This indicates that the “adhesive” friction mode *F_x_ ≈ τ*_0_*A* (4) is realized in the system. This subsection describes additional experiments in which the maximum force acting on the friction surface was varied over an even wider range. In this case, the experiments were carried out under the scenario with a tangential shear of the indenter along the *AB* trajectory (see Figure 4), and the experimental results are shown in Figure 8.

The dependencies shown in the figure are similar to those presented in Figure 5, but with the difference that, in the experiment shown in Figure 8, a larger indentation depth of *d*_max_ = 1.0 mm was used, while in Figure 5 the data for a smaller value of *d*_max_ = 0.6 mm are presented. To achieve a depth of *d*_max_ = 1.0 mm, the indenter was moved a distance of 25 mm in the tangential direction (along trajectory *AB* in Figure 4). In the BEM simulation, the results of which are shown by symbols in Figure 8, the maximal indentation depth (*d*_max_) was also 1.0 mm. Figure 8 shows the results of two experiments in which different materials, CRG N3005 and the softer elastomer CRG N0505, were used as the indentation substrate. In the BEM simulation, the indentation of the CRG N3005 material used the same elastic parameters as in Figure 5, i.e., *E *= 0.324 MPa, *ν *= 0.48, *h *= 5 mm, Δ*γ* = 0.0175 J/m^2^. For the softer elastomer CRG N0505, a lower value of elastic modulus *E *= 0.042 MPa was chosen, and all other parameters were similar to the material CRG N3005. Note that in Figure 8d only one calculated in BEM dependence (for material CRG N0505) is shown, because dependencies for both materials (CRG N3005 and CRG N0505) for indenter with the same radius (*R*) are almost the same. Each panel in Figure 8 shows only two experimental curves, one for each elastomer, since only one experiment was performed for each material (the repeatability of the experiments has been previously demonstrated in Figure 5 and Figure 7). It is worth noting that the *F_N_*(*t*) dependence shown in Figure 8a deviates from the simulation results regarding large *F_N_* forces. The deviation is caused due to the fact that the experimental setup used (see Figure 2) was designed for much smaller normal forces than those used in the experiment. Therefore, the device does not have sufficient rigidity to maintain a fixed indentation depth at high *F_N_* forces. Going into detail, it was in this particular experiment that a partial mechanical failure occurred at one corner of the glass plate that held the elastomer. As a result of the glass fracture, there was a change in the normal force dependence when the indenter was further indented. Nevertheless, despite the existing problem, in the experiment shown in Figure 8, the maximum normal force (*F_N_*) was of the order of 60 N, which is three times larger than the previously used maximum value of *F_N_* ≈ 20 N (see Figure 5). However, the dependence of *μ*(*t*) shown in Figure 8f, even over such a large load range, demonstrates a steady decrease in the friction coefficient as the *F_N_* force increases (at least up to the moment of the glass plate fracture). In this case, *μ*(*t*) does not reach a constant value, which confirms that there is no transition between “adhesive” *F_x_ = τ*_0_*A* (4) and “normal” *F_x_ = μF_N_* (3) friction, since over the entire range of normal forces the friction force (*F_x_*) is satisfactorily described by Equation (4).

The results for the softer CRG N0505 elastomer, also presented in Figure 8, show a much lower critical shear stress value of *τ*_0_ *≈* 7 kPa, resulting in a much lower friction force (*F_x_*) compared to the stiffer material. Note that material CRG N0505 shows significantly lower value of the specific work of adhesion Δ*γ* in comparison with harder CRG N3005 [42]. However, despite this, CRG N0505 shows better contact propagation due to adhesion in the normal direction, since it is softer and more deformable. Therefore, the contact area (*A*) increases faster in the case of softer material and takes on a larger value at the maximum indentation depth (*d*_max_) (see Figure 8d). It is also interesting that, in the case of softer material, the dependence of *A*(*t*), as shown in Figure 8d, undergoes abrupt changes both during indentation and at detachment. This is due to the propagation of elastic waves in the contact, which is well visualized in Supplementary Video S3. Such waves are called Schallamach waves [48,49] and represent one of the least-studied and intriguing processes in modern friction physics.

Figure 6c shows the dependence of the angle *α_F_* between the directions of indenter motion and the resulting tangential force (*F_t_*) acting in the contact plane. The above dependencies show that the angle *α_F_* is small, indicating that the experiments were set up correctly. From the comparison of all the data in Figure 6, it can be seen that only in the case of elastomer torsion (Figure 6b) the angle *α_F_* takes significantly different values from zero, which can affect the experimental results. However, when solving the problems posed in the proposed work, the value of *α_F_* does not have a determining influence, since the friction coefficient (*μ*) is calculated using the resultant tangential force (*F_t_*) (20), which also takes into account the lateral component of the friction force (*F_y_*).

## 5. Discussion

**Figure 9:**
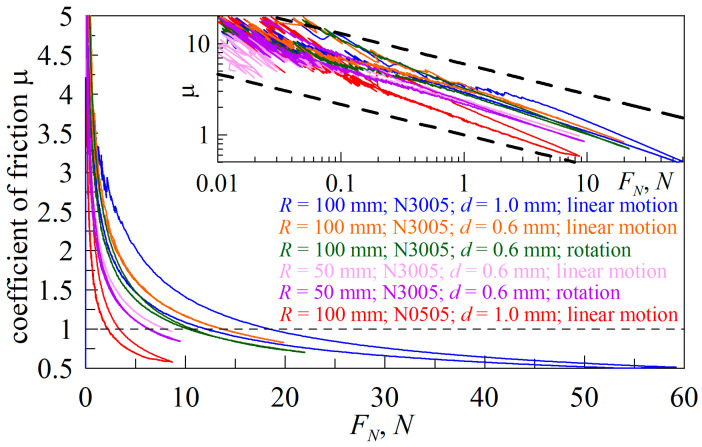
Dependencies of the friction coefficient (*μ*) on the external normal force (load) (*F_N_*) for all experiments conducted within the framework of this work. All dependencies are shown in different colors; the conditions of the corresponding experiments are briefly described with the same colors. The inset to the figure shows the same dependences, only in logarithmic coordinates, and the dashed lines in the inset show two dependences, $\mu \propto {\left(F_{N}\right)}^{-1/3}$, which serve to demonstrate the friction law (18).

Figure 9 shows the dependences of the friction coefficient (*μ*) on the normal force (*F_N_*) for all experiments described in the text above.

All dependencies are presented in different colors, and the same color in the figure panel summarizes information about the experimental conditions: indenter radius (*R*), elastomer type (CRG N3005 or CRG N0505), maximum indentation depth (*d*_max_) (0.6 mm or 1.0 mm), and if a linear or rotational motion scenario was used (see trajectories *AB* and *A’B’* in Figure 4). Figure 9 shows six dependencies that correspond to the data shown in Figure 5, Figure 7 and Figure 8. In order not to overload the figure, for experiments in which several indentation cycles were performed (these are Figure 5 and Figure 7), only the first cycle is shown. Thus, Figure 9 contains the results of all the experiments described in the paper performed under different conditions. Note that the *μ*(*F_N_*) dependences shown in the figure contain results for both the indentation and detachment phases. Often in these phases different dependences, such as *μ*(*F_N_*), are realized, which leads to an apparent hysteresis of the friction coefficient during loading and unloading of the contact. Hysteresis is well visualized in the lower curve corresponding to the CRG N0505 material. The upper part of the curve corresponds to the detachment phase, i.e., larger values of the friction coefficient (*μ*) at a given normal force (*F_N_*) are realized. There may be several reasons for this behavior, one of them is the hysteresis of the adhesive contact area, which consists of the fact that, in the detachment phase, the contact area is always larger than the area that corresponds to the same indentation depth in the indentation phase. If the area (*A*) is larger, the resulting friction force (*F_x_ = τ*_0_*A*) (4) is also larger, which can lead to an increase in the friction coefficient. Note that the area hysteresis during loading/unloading of the contact is also observed in non-adhesive contacts, as experimentally shown in [27]. Also, the discussed increase in the friction coefficient (*μ*) can be related to viscoelasticity, since the indenter was pulled off at a bigger normal velocity than it was indented (see Section 3.2). Another explanation might be the effect of contact hardening with time that was discussed in our previous work [23], in which normal indenter detachment experiments were also performed in a tangentially loaded contact.

The inset of Figure 9 shows the same dependencies as in the main panel of the figure, only on a logarithmic scale. Here, bold dashed lines show the dependences $\mu \propto {1/F_{N}}^{1/3}$, corresponding to the friction law (17). Note that, according to Equation (17), under otherwise equal conditions, the friction coefficient (*μ*) increases with the increase in the indenter radius (*R*), which is observed in Figure 9 when comparing experiments with indenters of different radii. The experimental dependencies are generally well described by the law $\mu \;\propto {1/F_{N}}^{1/3}$, as indicated in the inset of Figure 9. Although in some situations, such as the experiment with the CRG N0505 elastomer (bottom curve), the coefficient of friction decreases with the increasing force of *F_N_* faster than what is given by Equation (17). However, even in this case, the dependence of *μ*(*F_N_*) follows a power law, since in logarithmic coordinates it represents a straight line.

The reason for the deviation of *μ*(*F_N_*) from Equation (17), as observed in Figure 9, is that, in the experiment, a sheet of rubber with a limited thickness of *h *= 5 mm is used as a substrate upon which indentation is performed. As it is known, as the thickness of elastomer decreases, its stiffness increases. Therefore, for the same normal force (*F_N_*), a thinner layer corresponds to a smaller indentation depth (*d*) and, as a result, a smaller contact area (*A*), and thus a smaller friction force (*F_x_ = τ*_0_*A*) (4). As a result, for thin layers, the friction coefficient *μ = F_x_/F_N_* decreases faster as the *F_N_* force increases, as demonstrated in our experiments. The described effect of reducing the friction coefficient by coating hard surfaces with layers of softer materials is well known and actively used in the industry [24,39,50].

**Figure 10:**
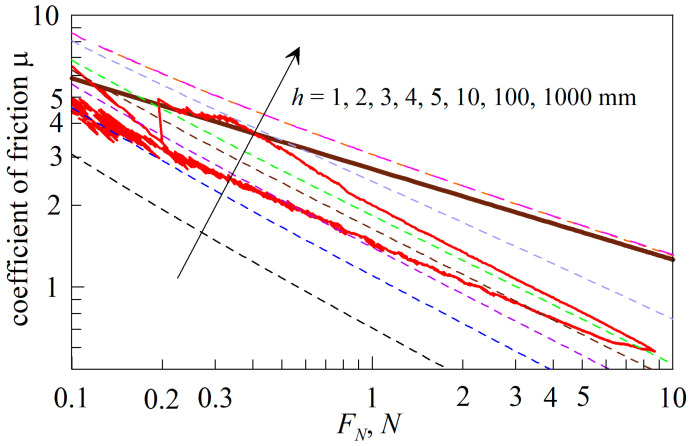
Dependences of the friction coefficient (*μ*) on the external normal force (load) (*F_N_*). The dashed dependencies show the results of BEM simulations when indenting an indenter with a radius of *R* = 100 mm into an elastic substrate with parameters *E* = 0.042 MPa, *ν* = 0.48, Δ*γ* = 0.0175 J/m^2^, which correspond to the material CRG N0505. The dashed curves from bottom to top (8 curves in total) correspond to cases with increasing elastomer thickness (*h*); the thickness values are shown in the panel of the figure. The brown bold line shows the dependence following a power law (17), obtained at *τ*_0_ = 7 MPa and the elastic parameters mentioned above. The red solid line of smaller thickness shows the results of the experiment on the indentation into CRG N0505 material (see Figure 8).

Since the dependence of the friction coefficient on the thickness of the elastomer layer is of practical importance, we performed additional BEM calculations, the results of which are shown in Figure 10. From the figure, it follows that, as the elastomer thickness (*h*) decreases, the friction coefficient (*μ*) decreases also. The two upper curves corresponding to the values of thicknesses (*h *= 100 mm and *h *= 1000 mm) overlap, since at these elastomer thicknesses the half-space conditions are already fulfilled and a further increase in the thickness of *h* will not lead to a visible change in the results. The solid straight line in Figure 10 shows the theoretical dependence for the half-space (17). It follows from the figure that as the normal force (*F_N_*) increases, the obtained solutions for large layer thicknesses (*h *= 100 mm and *h *= 1000 mm), which correspond to the half-space, approach this theoretical straight line. The deviation of the simulation results from the theoretical estimate of (17) at small normal loads is due to assuming the absence of adhesion in the normal direction during the derivation of Equation (17). At the same time, the simulation considers adhesive contact, in which the contact area is larger and thus the friction force (*F_x_*), which is proportional to the contact area, is higher. In general, Figure 10 demonstrates that the dependence of the friction coefficient on the normal load at different elastomer thicknesses can be described by the power law $\mu \propto {1/F_{N}}^{\alpha }$, where the exponent *α* takes a minimum value of *α *= 1/3 in the case of the half-space and increases with decreasing elastomer thickness (*h*). This behavior is evident for the reasons described above and consistent with the experimental results, which are also shown in Figure 10. Thus, the change in the value of the degree exponent *α*, determining the rate of decrease in the friction coefficient (*μ*) with the external load *F_N_*, can be caused not only by a change in the contact geometry (see Equation (13) and the paragraph describing it), but also by the effect of the thickness (*h*) of the elastomer layer.

**Figure 11:**
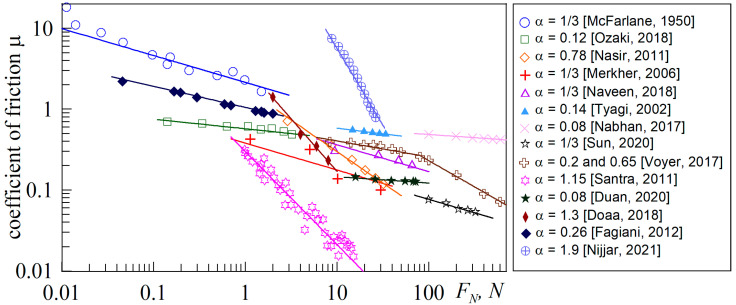
Dependences of the friction coefficient (*μ*) on the external normal force (*F_N_*). Symbols represent experimental data from [26,32,51,52,53,54,55,56,57,58,59,60,61,62], solid lines show the exponent approximations $\mu \propto {1/F_{N}}^{\alpha }$, where the value of the exponent *α* for all straight lines are given in the figure panel with a reference to the source with the corresponding experimental data.

Figure 11 shows the experimental dependencies of the friction coefficient (*μ*) on the external load (*F_N_*) taken from the works of various authors [26,32,51,52,53,54,55,56,57,58,59,60,61,62]. Here, the upper dependences are the data from [32], previously discussed above and shown in Figure 1. To avoid misunderstandings, we note that in Figure 1 the mass in grams was plotted along the abscissa axis because the original data from [32] was shown, while Figure 11 shows the dependence of the friction coefficient on the external force expressed in Newtons.

The only criterion for selecting the dependences shown in Figure 11 was that the experimentally measured friction coefficient (*μ*) should decrease with the increase in the external load. Therefore, the dependencies shown in Figure 11 correspond to experiments that were performed with different materials and under different conditions, including different contact geometries, indenter velocities, etc. However, the general behavior observed here is that all *μ*(*F_N_*) dependencies are following a power law. That means all dependencies are described by the functions $\mu \propto {1/F_{N}}^{\alpha }$, represented in the figure by straight lines, with the values of the exponent α also given in the figure. It is worth noting that the data in Figure 11 are given in a fairly wide range of external forces (*F_N_*) from 0.01 N to 600 N, and for all cases a power-law decrease in the friction coefficient (*μ*) is observed. Moreover, the exponent *α* varies in different experiments in a fairly wide range—from 0.08 [55,59] to 1.9 [62].

We note two interesting features that follow from Figure 11. The first one is that, in some cases, the exponent *α <* 1/3 [26,54,55,57,59,61], indicating the realization of experimental conditions or even a friction mechanism different from those considered in the present work. In fact, as demonstrated in Figure 10, when the frictional force is proportional to the contact area (adhesive contact) and the indenter is spherical, the minimum possible value of *α *= 1/3 is achieved in the half-space limit. This value of α is realized in 4 out of 14 experiments [32,52,53,56], the data of which are shown in Figure 11. However, we should also keep in mind that another reason for the change in the decreasing rate of the friction coefficient may be the deviation of the indenter geometry from the spherical one (see Equation (13) and its explanations). Indeed, in some experiments, and in the data shown in Figure 11, the geometry of the experiment deviated strongly from the case of contact of a sphere with a smooth surface.

The second point worth noting is the data from [57], which investigated friction over a wide range of loads, from 10 N to 600 N. Figure 11 shows that, initially, with increasing normal load, the friction coefficient decreases rather slowly with an exponent of α = 0.2, but when the force exceeds a critical value of *F_N_ > F_C_ ≈* 80 N, the friction coefficient continues to decrease much faster, which corresponds to a greater value of α = 0.65. Thus, in [57], a regime is observed in which an increase in the load on the friction surface leads to a change in the friction mode.

## 6. Conclusions

In the proposed work, adhesive contact between a spherical steel indenter and a much softer elastomer is studied experimentally. In this case, the friction force is proportional to the contact area, resulting in a friction coefficient that decreases in accordance with a power law and an increasing external load on the contacting surfaces. The experimental results are consistent with both the theoretical predictions and the simulations carried out in the work. The main goal of the proposed work was to study the presence of a transition between the modes of “adhesive” friction, in which the friction force is proportional to the contact area, and a “normal” friction mode, in which the familiar Amonton’s law is satisfied. The presence of such a transition with an increasing external load on the friction surface is stated in some classical works; however, in our system, such a transition was not clearly detected. Moreover, it was demonstrated that some of the results of other authors showing the transition between “adhesive” and “normal” friction can be described within the framework of the “adhesive” friction regime over the entire range of normal loads on the friction surface. When analyzing experimental data from some works by other authors, it was found that in experiments where the friction coefficient decreases with an increase in the external load, this decrease is most often described by a power function, with the exponent varying widely. The value of the exponent, which determines the rate of decrease in the friction coefficient with an increase in the external load, depends on the thickness of the layer with which the indenter is in contact, as well as on the geometric shape of the indenter. If the indenter has a conical shape, the friction coefficient becomes independent of the normal force, although the friction force remains proportional to the contact area. Moreover, it is possible to produce indenters with shapes for which the friction coefficient increases as the load increases, as shown analytically. We are planning to investigate such cases experimentally in the future.

## Data Availability

The datasets generated for this study are available upon request from the corresponding author.

## Supplementary Materials

The following supporting information can be downloaded at: https://www.mdpi.com/article/10.3390/biomimetics9010052/s1. Video S1: Experiments performed according to the scenario, a schematic of which is shown by trajectory *AB* in Figure 4. Spherical steel indenter was immersed to a depth of *d*_max_ = 0.6 mm into a layer of TARNAC CRG N3005 rubber with a thickness of *h* = 5 mm. In the indentation phase, the indenter was shifted at an angle *α* to the elastomer surface with the velocities *v_n_* = 0.2 μm/s in normal and *v_t_* = 5 μm/s in tangential directions, which gives the value of the angle as *α* = arctan (0.2/5) ≈ 2.29°. The video shows a series of two experiments with indenters of different radii: *R* = 100 mm and *R* = 50 mm. After the indentation to the maximum depth (*d*_max_), the indenter was pulled off the elastomer in the normal direction with a velocity of *v* = 1 μm/s. Separate panels in the video show the time dependencies of the normal (*F_N_*) and tangential (*F_x_*) forces, contact area (*A*), average tangential stresses <*τ*> = *F_x_*/*A* and formally calculated friction coefficient <*μ*> = *F_x_*/*F_N_*. In addition, the lower left panel shows the evolution of the contact zone; it also shows the current values of the indentation depth (*d*), the tangential shift of the indenter (*x*), and the time (*t)* that has passed since the beginning of the indentation. The video relates to Figure 5 in the article. Video S2: It is similar to the Video S1 experiments, but with one difference: that, in this case, the indenter was moved only in the normal direction and the elastomer layer was rotated with fixed angular velocity (direction of elastomer layer rotation is shown in Video by arrow). The distance between the axis of the indenter and the rotation axis of the elastomer was *L* = 26 mm. Schematically, the experiment is shown by trajectory *A’B’* in Figure 4 (full description of the experimental procedure can be found in the article text with the explanation of Figure 4). The video relates to Figure 7 in the article. Video S3: It is similar to the Video S1 experiments, but with bigger values of maximum indentation depth (*d*_max_ = 1.0 mm) and, as a result, a greater length of the path *AB* = 25 mm, which is shown in Figure 4. The video shows a series of two experiments with the indenter of the same radius (*R* = 100 mm), but with two different elastomers: CRG N3005 as in previous experiments and the much softer material CRG N0505. The video relates to Figure 8 in the article.

## References

[1] Alberts B., Johnson A., Lewis J., Morgan D., Raff M., Roberts K., Walter P. (2014). Molecular Biology of the Cell.

[2] Gumbiner B.M. (1996). Cell Adhesion: The Molecular Basis of Tissue Architecture and Morphogenesis. Cell.

[3] Linke D., Goldman A. (2011). Bacterial Adhesion—Chemistry, Biology and Physics.

[4] Gao H., Wang X., Yao H., Gorb S., Arzt E. (2005). Mechanics of hierarchical adhesion structures of geckos. Mech. Mater..

[5] Lutz T.M., Kimna C., Casini A., Lieleg O. (2022). Bio-based and bio-inspired adhesives from animals and plants for biomedical applications. Mater. Today Bio.

[6] Dirks J.-H., Federle W. (2011). Fluid-based adhesion in insects—Principles and challenges. Soft Matter.

[7] Huber G., Mantz H., Spolenak R., Mecke K., Jacobs K., Gorb S.N., Arzt E. (2005). Evidence for capillarity contributions to gecko adhesion from single spatula nanomechanical measurements. Proc. Natl. Acad. Sci. USA.

[8] Higham T.E., Russell A.P., Niewiarowski P.H., Wright A., Speck T. (2019). The Ecomechanics of Gecko Adhesion: Natural Surface Topography, Evolution, and Biomimetics. Commun. Integr. Biol..

[9] Li J., Zhang Y., Liu S., Liu J. (2018). Insights into adhesion of abalone: A mechanical approach. J. Mech. Behav..

[10] Xi P., Ye S., Cong Q. (2023). Abalone adhesion: The role of various adhesion forces and their proportion to total adhesion force. PLoS ONE.

[11] Federle W., Labonte D. (2019). Dynamic biological adhesion: Mechanisms for controlling attachment during locomotion. Phil. Trans. R. Soc..

[12] Wang Z., Xing Q., Wang W., Ji A., Dai Z. (2018). Contribution of friction and adhesion to the reliable attachment of a gecko to smooth inclines. Friction.

[13] Jagota A., Hui C.Y. (2011). Adhesion, friction, and compliance of bio-mimetic and bio-inspired structured interfaces. Mater. Sci. Eng. R Rep..

[14] Niederegger S., Gorb S.N. (2006). Friction and adhesion in the tarsal and metatarsal scopulae of spiders. J. Comp. Physiol. A.

[15] Waters J.F., Guduru P.R. (2010). Mode-mixity-dependent adhesive contact of a sphere on a plane surface. Proc. R. Soc. A.

[16] Peng X., Zhang Z. (2019). Improvement of paint adhesion of environmentally friendly paint film on wood surface by plasma treatment. Prog. Org. Coat..

[17] Schmitt P., Eberlein D., Ebert C., Tranitz M., Eitner U., Wirth H. (2013). Adhesion of Al-metallization in Ultra-Sonic Soldering on the Al-Rear Side of Solar Cells. Energy Procedia.

[18] Wang H.Y., Liu L.M. (2014). Analysis of the influence of adhesives in laser weld bonded joints. Int. J Adhes..

[19] Ma C., Sun J., Li B., Feng Y., Sun Y., Xiang L., Wu B., Xiao L., Liu B., Petrovskii V.S. (2021). Ultra-strong bio-glue from genetically engineered polypeptides. Nat. Commun..

[20] Przywara M., Dürr R., Otto E., Kienle A., Antos D. (2021). Process Behavior and Product Quality in Fertilizer Manufacturing Using Continuous Hopper Transfer Pan Granulation-Experimental Investigations. Processes.

[21] Pizzi A., Mittal K.L. (2017). Handbook of Adhesive Technology.

[22] Derjaguin B. (1934). Molekulartheorie der äußeren Reibung. Z. Physik.

[23] Lyashenko I.A., Popov V.L., Borysiuk V. (2023). Indentation and Detachment in Adhesive Contacts between Soft Elastomer and Rigid Indenter at Simultaneous Motion in Normal and Tangential Direction: Experiments and Simulations. Biomimetics.

[24] Bowden F.P., Tabor D. (1950). The Friction and Lubrication of Solids.

[25] Yoshizava H., Chen Y.-L., Israelachvili J. (1993). Fundamental mechanisms of interfacial friction. 1. Relation between adhesion and friction. J. Phys. Chem..

[26] Ozaki S., Mieda K., Matsuura T., Maegawa S. (2018). Simple Prediction Method for Rubber Adhesive Friction by the Combining Friction Test and FE Analysis. Lubricants.

[27] Liang X.M., Xing Y.Z., Li L.T., Yuan W.K., Wang G.F. (2021). An experimental study on the relation between friction force and real contact area. Sci. Rep..

[28] Otsuki M., Matsukawa H. (2013). Systematic Breakdown of Amontons’ Law of Friction for an Elastic Object Locally Obeying Amontons’ Law. Sci. Rep..

[29] Popov V.L., Li Q., Lyashenko I.A., Pohrt R. (2021). Adhesion and friction in hard and soft contacts: Theory and experiment. Friction.

[30] Lyashenko I.A., Popov V.L. (2022). The influence of adhesion on rolling and sliding friction: An experiment. Tech. Phys..

[31] Lyashenko I.A., Popov V.L., Borysiuk V. (2023). Experimental Verification of the Boundary Element Method for Adhesive Contacts of a Coated Elastic Half-Space. Lubricants.

[32] McFarlane J.S., Tabor D. (1950). Relation between friction and adhesion. Proc. R. Soc. Lond. A.

[33] Homola A.M., Israelachvili J.N., McGuiggan P.M., Gee M.L. (1990). Fundamental experimental studies in tribology: The transition from “interfacial” friction of undamaged molecularly smooth surfaces to “normal” friction with wear. Wear.

[34] Johnson K.L., Kendall K., Roberts A.D. (1971). Surface energy and the contact of elastic solids. Proc. R. Soc. Lond. A.

[35] Hertz H. (1881). Ueber die Berührung fester elastischer Körper. J. Reine Angew. Math..

[36] Popov V.L., Hess M. (2014). Method of dimensionality reduction in contact mechanics and friction: A users handbook. I. Axially-Symmetric Contacts. Facta Univ. Ser. Mech. Eng..

[37] Davis P.J. (1959). Leonhard Euler’s Integral: A Historical Profile of the Gamma Function. Am. Math. Mon..

[38] Roberts E.W. (1989). Ultralow friction films of MoS_2_ for space applications. Thin Solid Films.

[39] Martin J.-M., Erdemir A., Martin J.-M. (2007). 13—Superlubricity of Molybdenum Disulfide. Superlubricity.

[40] Müser M.H., Gnecco E., Meyer E. (2015). Theoretical Studies of Superlubricity. Fundamentals of Friction and Wear on the Nanoscale.

[41] Lyashenko I.A., Popov V.L., Pohrt R., Borysiuk V. (2023). High-Precision Tribometer for Studies of Adhesive Contacts. Sensors.

[42] Lyashenko I.A., Popov V.L. (2021). Hysteresis in an adhesive contact upon a change in the indenter direction of motion: An experiment and phenomenological model. Tech. Phys..

[43] Pohrt R. (2020). Friction Influenced by Vibrations: A Refined Contact-Mechanics View on Lateral and Rotational Oscillations. Front. Mech. Eng..

[44] Li Q., Pohrt R., Lyashenko I.A., Popov V.L. (2020). Boundary element method for nonadhesive and adhesive contacts of a coated elastic half-space. Proc. Inst. Mech. Eng. Part J J. Eng. Tribol..

[45] Greenwood J.A. (2017). Reflections on and Extensions of the Fuller and Tabor Theory of Rough Surface Adhesion. Tribol. Lett..

[46] Lyashenko I.A., Popov V.L. (2020). The effect of contact duration and indentation depth on adhesion strength: Experiment and numerical simulation. Tech. Phys..

[47] Lyashenko I.A., Pohrt R. (2020). Adhesion between rigid indenter and soft rubber layer: Influence of roughness. Front. Mech. Eng..

[48] Schallamach A. (1971). How does rubber slide?. Wear.

[49] Barquins M. (1985). Sliding Friction of Rubber and Schallamach Waves—A Review. Mater. Sci. Eng..

[50] Chen Z. (2019). Friction Reduction Effect of Soft Coatings. J. Tribol..

[51] Nasir R.M., El-Tayeb N.S.M. (2011). Surface morphology, mechanical and tribological properties of blended deproteinized natural and polyisoprene rubbers. J. Thermoplast. Compos. Mater..

[52] Merkher Y., Sivan S., Etison I., Maroudas A., Halperin G., Yosef A. A rational friction test using human cartilageon-cartilage arrangement. Proceedings of the AITC-AIT 2006 International Conference on Tribology.

[53] Naveen G.J., SampathKumaran P., Badrinath P., Vynatheya S., Sailaja R.R.N., Seetharamu S., Deepthi M.V., Niranjan H.B. (2018). Role of Graphene oxide and addition of MoS2 in HDPE matrix for improved tribological properties. IOP Conf. Ser. Mater. Sci. Eng..

[54] Tyagi R., Nath S.K., Ray S. (2002). Effect of martensite content on friction and oxidative wear behavior of 0.42 Pct carbon dual-phase steel. Metall. Mater. Trans. A.

[55] Nabhan A., El-Sharkawy M.R., Ali W.Y. Proper Selection of Floor Materials for Wheelchair Users. Proceedings of the New Vision to Challenge Disabilities International Conference.

[56] Sun K., Peng W., Wei B., Yang L., Fang L. (2020). Friction and Wear Characteristics of 18Ni(300) Maraging Steel under High-Speed Dry Sliding Conditions. Materials.

[57] Voyer J., Ausserer F., Klien S., Velkavrh I., Diem A. (2017). Reduction of the Adhesive Friction of Elastomers through Laser Texturing of Injection Molds. Lubricants.

[58] Santra T.S., Bhattacharyya T.K., Tseng F.G., Barik T.K. Diamond-Like Nanocomposite (DLN) Films for Microelectro-Mechanical System (MEMS). Proceedings of the IJCA International Symposium on Devices MEMS, Intelligent Systems & Communication (ISDMISC).

[59] Duan Y., Qu S., Yang C., Li X., Liu F. (2020). Drop Tower Experiment to Study the Effect of Microgravity on Friction Behavior: Experimental Set-up and Preliminary Results. Microgravity Sci. Technol..

[60] Doaa G.Z., Meshref A.A., Mazen A.A. (2018). Friction and wear of epoxy reinforced by iron nano particles. EGTRIB J. J. Egypt. Soc. Tribol..

[61] Fagiani R., Massi F., Chatelet E., Costes J.P., Berthier Y. (2012). Contact of a Finger on Rigid Surfaces and Textiles: Friction Coefficient and Induced Vibrations. Tribol. Lett..

[62] Nijjar S., Sudhakara P., Sharma S., Saini S., Teklemariam A., Mariselvam V., Sampath S.K., Song J.I. (2021). Influence of Alkali Treatment and Maleated Polypropylene (MAPP) Compatibilizer on the Dry-Sliding Wear and Frictional Behavior of Borassus Fruit Fine Fiber (BFF)/Polypropylene (PP) Polymer Composites for Various Engineering Applications. Adv. Mater. Sci. Eng..

